# The impact of an oral glucose load on IFN-γ-release in persons infected with *Mycobacterium tuberculosis*

**DOI:** 10.1186/s12879-024-09920-x

**Published:** 2024-09-30

**Authors:** Hans Johan Niklas Lorentsson, Christina Reinholdt Clausen, Daniel Faurholt-Jepsen, Katrine Bagge Hansen, Christian Ritz, Sidse Graff Jensen, Erik Michael Rasmussen, Anja Jørgensen, Troels Lillebaek, Filip Knop, Pernille Ravn

**Affiliations:** 1 Unit of Infectious Diseases, Department of Medicine, Herlev and Gentofte Hospital, University of Copenhagen, Hellerup, Denmark; 2 Center for Clinical Metabolic Research, Herlev and Gentofte Hospital, University of Copenhagen, Hellerup, Denmark; 3 Department of Diabetes Treatment, Steno Diabetes Center Copenhagen, Herlev, Denmark; 4 Department of Infectious Diseases, Rigshospitalet, University of Copenhagen, Copenhagen, Denmark; 5 Department of Clinical Medicine, Faculty of Health and Medical Sciences, University of Copenhagen, Copenhagen, Denmark; 6 International Reference Laboratory of Mycobacteriology, Statens Serum Institut, Copenhagen, Denmark; 7 National Institute of Public Health, University of Southern Denmark, Copenhagen, Denmark; 8 Section of Respiratory Diseases, Department of Medicine, Herlev and Gentofte Hospital, University of Copenhagen, Hellerup, Denmark; 9 Global Health Section, Department of Public Health, University of Copenhagen, Copenhagen, Denmark; 10 Currently Employed at Novo Nordisk A/S, Søborg, Denmark

**Keywords:** Interferon-γ-release, Tuberculosis disease, Tuberculosis infection, Oral glucose tolerance test

## Abstract

**Background and objective:**

To diagnose tuberculosis infection (TBI), whole blood is incubated with *M.tuberculosis* (*Mtb*)-specific peptides and the release of interferon-γ (IFN-γ) is measured in IFN-γ-release assays (IGRAs). Hyperglycaemia and fluctuations in blood glucose may modulate IFN-γ-release. Here, we investigated if glucose intake affects IFN-γ-release or IGRA results in IGRAs taken during an oral glucose tolerance test (OGTT).

**Methods:**

Persons with TB disease (TB) or TBI underwent a standard 75-g OGTT at the start and end of treatment for TB or TBI. Blood for the IGRA QuantiFERON-TB Gold Plus (QFT) containing *Mtb*-specific tubes (TB1 and TB2), a non-specific mitogen tube (MIT) and an empty control tube (NIL) was drawn at sample-timepoints -15 (baseline), 60, 90, 120 and 240 min during the OGTT. Blood glucose was measured in parallel at all timepoints. IFN-γ-release (after subtraction of NIL) at each timepoint was compared with baseline using linear-mixed-model analysis.

**Results:**

Twenty-four OGTTs from 14 participants were included in the final analysis. Compared to baseline, IFN-γ-release was increased at sample-timepoint 240 min for TB1; geometric mean (95% confidence interval) 3.0 (1.5–6.2) vs 2.5 (1.4–4.4) IU/mL (*p* = 0.047), and MIT; 182.6 (103.3–322.9) vs 146.0 (84.0–254.1) IU/mL (*p* = 0.002). Plasma glucose levels were not associated with IFN-γ-release and the QFT test results were unaffected by the OGTT.

**Conclusion:**

Ingestion of glucose after a 10-h fast was associated with increased IFN-γ-release after 240 min in the MIT tube. However, there was no association between plasma glucose levels at the QFT sampling timepoint and IFN-γ-release. Furthermore, the QFT test results were not affected by glucose intake. The overall effect of an OGTT and prevailing plasma glucose levels on IFN-γ-release in IGRAs seem limited.

**Trial registration:**

Trial registration ID: NCT04830462 (https://clinicaltrials.gov/study/NCT04830462). Registration date: 05-Apr-2021.

**Supplementary Information:**

The online version contains supplementary material available at 10.1186/s12879-024-09920-x.

## Introduction

Tuberculosis disease (TB), caused by bacteria from the *Mycobacterium tuberculosis* complex, is a global health problem with 10.6 million new cases every year and 1.3 million deaths in 2022 [1].

An estimated quarter of the world’s population has a tuberculosis infection (TBI) which refers to a state of persistent immune response to stimulation by *Mycobacterium tuberculosis* (*Mtb*) antigens without evidence of clinical TB [1]. The bacteria can remain dormant for decades but in some cases, if the immune system is impaired, the infection can progress to TB resulting in subsequent spread of the bacteria and new cases of TB and TBI.

The number of persons living with diabetes mellitus (DM) is estimated to increase from 537 million in 2021 to 783 million in 2045, and the majority with DM live in low- and middle-income countries [2]. Many of these countries are also burdened with TB which is worrying as DM increases the risk of infections and impairs the immune system [1–3]. Accordingly, data from meta analyses show that persons with DM are at least 1.5 times more likely to present with TB or TBI and in 2000, 14.8% of all TB cases in India could be attributed to DM [4–6]. It has even been suggested that the impact of DM on TB rates could rival or surpass that of human immunodeficiency virus (HIV) [7]. DM is not only associated with increased risk of TB but also with TB severity, treatment failure, drug resistance, and smear positivity which all together lead to higher morbidity, mortality and increased spread of TB [3–5]. The cause of this association between TB and DM remains elusive but reduced immunity due to impaired cytokine signalling, compromised barrier function, dysfunctional macrophages, neutrophils, and leukocytes have all been implied as possible mechanisms [3].

Screening and prophylactic treatment are key elements to reduce the burden of TB and in many parts of the world, interferon-γ (IFN-γ)-release assays (IGRAs) such as the QuantiFERON-TB Gold Plus (QFT) are used for TBI screening. The principle behind the test is that T lymphocytes (T-cells) from *Mtb* sensitised persons express IFN-γ when stimulated with *Mtb*-specific antigens. The test requires a functional immune response as reflected by the compromised test performance in persons with severe immune deficiency due to HIV [8] or immunosuppressive treatment [9, 10]. The effect of DM on IGRAs as a test for TBI is not settled. Some studies find that DM and pre-DM are associated with an increased number of inconclusive and false negative QFT results [11, 12] while others report no effect on the test performance [13–15].

The impact of DM on quantitative IFN-γ-release is also debated with studies showing increased, decreased and unaffected IFN-γ-release in response to *Mtb*-specific and non-specific antigen stimuli [11, 12, 14, 16–21]. Differences in study populations and ethnicities have been suggested as reasons for the diverging results but these are unlikely the sole explanations [22]. Many of the studies measured fasting blood glucose in order to assess DM status but it was not reported if the QFT was taken in a fasted or fed state [11, 12, 14, 16–20]. This might be of importance as acute changes in blood glucose can alter the levels of cytokines [23, 24]. IGRAs such as the QFT could therefore be affected by glucose fluctuations and the diverging results may be driven by systematic differences in the participants’ prandial status. We hypothesise that IFN-γ-release is affected by glucose intake which could affect QFT test performance. The aim for this study was to investigate if an oral glucose load affects quantitative IFN-γ-release or the test result of the QFT.

## Methods

### Study design and participants

This is a sub-study to one observational study on TB and one study on TBI treatment (https://clinicaltrials.gov/study/NCT04830462). Both studies addressed the effect of TB and TBI treatments on glucose metabolism using oral glucose tolerance tests (OGTTs) conducted at the start and end of treatment. For the purpose of this sub-study, QFT samples were obtained together with the glucose samples at five timepoints during the OGTTs. Participants were included from the TB outpatient clinic at Herlev and Gentofte Hospital, Denmark from April 2021 to September 2022. Inclusion criteria were: planned treatment for TB or TBI, age above 18 years and signed informed consent. Exclusion criteria were: known immunosuppression (e.g. HIV, steroid treatment within 14 days before inclusion, ongoing chemotherapy, ongoing immunomodulating treatment, and splenectomy), contraindication to the antibiotics rifampicin and isoniazid (e.g. allergy), active liver disease, severe inflammatory or rheumatological diseases with immune activation and need for prolonged systemic treatment, active cancer, pregnancy, type 1 DM, recent antibiotic treatment (> 2 days), or severe infection within 14 days before enrolment (TBI only).

### Tuberculosis diagnosis and treatment

TB and TBI treatment and diagnosis were performed at the TB outpatient clinic by the attending physician according to global and local guidelines [25]. TBI cases were defined by a positive QFT at the TB clinic without any clinical or radiological signs of TB. TB cases were defined by either positive *Mtb* culture, positive *Mtb* DNA polymerase chain reaction or clinical and radiological signs of TB with significant improvement after treatment.

### Study procedures

Participants were asked to avoid exercise, coffee and alcohol 24 h before the OGTT and met after a 10-h overnight fast. Participants with DM were asked to pause their daily DM treatment two days before the OGTT and weekly DM treatment one week before the OGTT. Seventy-five grams of glucose was dissolved in 300 mL of water and ingested within five minutes at timepoint zero minutes. QFT and glucose samples were taken at minute -15 (baseline), 60, 90, 120 and 240 after glucose intake. The procedure was performed before and after treatment of TB or TBI.

### Data collection and sample handling

Clinical information (e.g. Charlson comorbidity scoring, country of origin, alcohol and tobacco use) was obtained at inclusion and/or by review of the electronic patient record [26]. Blood was collected directly into the QFT tubes (QIAGEN, Germantown, MD, USA) which consist of a negative control (NIL) tube, two tubes (TB1 and TB2) with *Mtb*-specific peptides and a mitogen (MIT) tube with the nonspecific stimulant phytohaemagglutinin-P [27]. Handling was performed according to the manufacturer’s instructions [27]. Briefly described, the tubes were inverted and rotated 10 times before placement in an incubator (INCU-Line® IL 10, Avantor, Radnor, PA, USA) within 5 min from blood draw. After 23 h incubation, samples were centrifuged for 15 min (2000 g, 4 °C) and plasma was stored at -80 °C until analysis. IFN-γ release was measured using enzyme-linked immunosorbent assays (ELISA) that were handled and analysed using the automated DYNEX DS2® (Dynex, Chantilly, Virginia, USA) according to the manufacturer’s instructions. Samples collected during the same OGTT were analysed on the same ELISA plate. Samples intended for plasma glucose analysis were drawn with a syringe and dispensed immediately into a microvette tube containing heparin and fluoride (Sarstedt, Nümbrecht, Germany). The samples were centrifuged and analysed directly using the glucose oxidase method (model 2900 STAT Plus analyser; YSI, Yellow Springs, Ohio, USA).

### Interpretation of QFT test results and dilution for quantitative analysis

Analysis of the QFT test results was performed according to the manufacturer’s instructions with the addition that samples where NIL was larger than TB1 or TB2 were excluded from the final analysis due to suspected sample error [27]. If all 5/5 QFT samples from an OGTT were negative, the participant was deemed not infected or seroreverted and samples from that OGTT were not included in the analysis. IFN-γ levels above 10 IU/ml were not quantifiable with our method and samples above 10 IU/ml were therefore diluted before quantitative analysis.

### Statistical analysis

NIL was subtracted from TB1, TB2 and MIT for all analysis apart from NIL analysis. Statistical analysis on the effect of the timepoint (60, 90, 120, 240 min) after oral glucose ingestion vs baseline (-15 min) on IFN-γ-release was performed with linear mixed-effect model analysis where timepoint and OGTT-session (before/after treatment) were treated as a fixed effects and participant ID as a random effect. Statistical analysis of the effect of plasma glucose levels on IFN-γ-release was performed with linear mixed-effect model analysis where plasma glucose, timepoint and OGTT-session were treated as fixed effects and participant ID as a random effect. *P* values were adjusted for multiple comparisons using the Bonferroni method. Adjusted *p* values below 0.05 were considered significant. Data are presented as geometrical means with 95% confidence intervals (CI). The statistical analysis was performed in R version 4.1.0.

## Results

A total of 14 participants were included in the final OGTT analysis. Ten participants contributed with two OGTTs and four contributed with one OGTT adding up to a total of 24 OGTTs (Supplementary Figure S1). Clinical characteristics of the participants are presented in Table 1.

**Table 1:** Baseline characteristics of the participants

	**Participants** **(*n* = 14)**
**Baseline characteristics**	
Male sex, n (%)	7 (50%)
Age (years)	50.3 (15.2)
Tuberculosis infection, n (%)	11 (79%)
Tuberculosis disease, culture positive, n (%)	2 (17%)
Tuberculosis disease, clinical diagnosis, n (%)	1 (7%)
Diabetes, n (%)	2 (17%)
Body mass index (kg/m^2^)	25.5 (3.8)
Alcohol use (units/week)	2.8 (4.4)
Current smokers, n (%)	1 (7%)
Former smokers, n (%)	5 (36%)
Charlson comorbidity score	1.3 (1.6)
Leukocyte count (10^9^/L)	5.5 (1.1)
C-reactive protein (mg/L)	9.0 (16.5)
Glycosylated haemoglobin (mmol/mol)	39.8 (8.1)
Country of origin – Europe, n (%)	4 (29%)
Country of origin – Asia, n (%)	6 (43%)
Country of origin – South America, n (%)	1 (7%)
Country of origin – Africa, n (%)	3 (21%)

Baseline characteristics of persons with tuberculosis disease or tuberculosis infection who participated in the study. Data are presented as n (%) or mean (standard deviation)

**Fig. 1:**
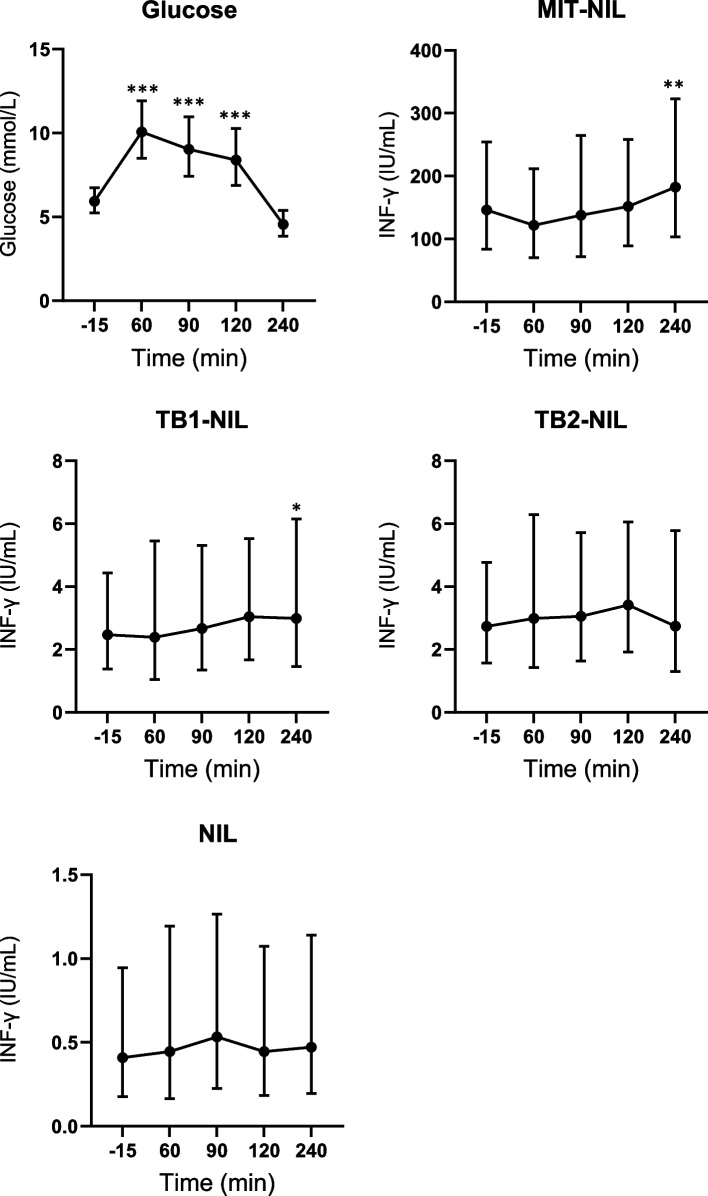
Plasma glucose levels and interferon-γ-release in samples taken at -15, 60, 90,120 and 240 min post a glucose load. Data are from 24 OGTTs from 14 participants presented as geometric means with 95% confidence intervals as error bars. Results from minute 60, 90, 120, and 240 were analysed vs. baseline (-15 min) using linear mixed-effect model analysis. The shown *p* values were adjusted using the Bonferroni method. NIL, negative control; MIT, mitogen; TB1, TB antigen tube 1; TB2, TB antigen tube 2. * *p* < 0.05, ** *p* < 0.005, *** p < 0.001

As visualised in Fig. 1 and summarised in Table 2, plasma glucose peaked at timepoint 60 min with a geometric mean of 10.1 (CI 8.5–11.9) mmol/L and returned to baseline values at timepoint 240 min. IFN-γ-release was increased at timepoint 240 min vs baseline for TB1 (3.0 (CI 1.5–6.2) vs 2.5 (CI 1.4–4.4) IU/mL, (*p* = 0.047)) and MIT (182.6 (CI 103.3–322.9) vs 146.0 (CI 84.0–254.1) IU/mL (*p* = 0.002)). TB2 was not affected by glucose intake.

**Table 2:** Plasma glucose levels and corresponding interferon-γ-release in samples taken at -15, 60, 90,120 and 240 min post a glucose load

	**-15 minutes**	**60 minutes**	**90 minutes**	**120 minutes**	**240 minutes**	***P* value (-15 vs 60)**	***P* value (-15 vs 90)**	***P* value (-15 vs 120)**	***P* value (-15 vs 240)**
Glucose (mmol/L)	**5.9 **(5.2–6.7)	**10.1 **(8.5–11.9)	**9.0 **(7.4–11.0)	**8.4 **(6.9–10.3)	**4.6 **(3.9–5.4)	< 0.001/ **< 0.001**	< 0.001/ **< 0.001**	< 0.001/** < 0.001**	0.016/ 0.375
NIL (IU/mL)	**0.4 **(0.2–0.9)	**0.4 **(0.2–1.2)	**0.5 **(0.2–1.3)	**0.4 **(0.2–1.1)	**0.5 **(0.2–1.1)	0.398/ 0.999	0.774/ 0.999	0.526/ 0.999	0.930/ 0.999
TB1-NIL (IU/mL)	**2.5 **(1.4–4.4)	**2.4 **(1.0–5.5)	**2.7 **(1.3–5.3)	**3.0 **(1.7–5.5)	**3.0 **(1.5–6.2)	0.467/ 0.999	0.139/ 0.999	0.267/ 0.999	0.002/ **0.047**
TB2-NIL (IU/mL)	**2.7 **(1.6–4.8)	**3.0 **(1.4–6.3)	**3.1 **(1.6–5.7)	**3.4 **(1.9–6.1)	**2.7 **(1.3–5.8)	0.026/ 0.600	0.024/ 0.549	0.065/ 0.999	0.079/ 0.999
MIT-NIL (IU/mL)	**146.0 **(84.0-254.1)	**121.9 **(70.2–211.9)	**137.8 **(71.9–264.4)	**151.7 **(89.1–258.3)	**182.6 **(103.3–322.9)	0.575/ 0.999	0.290/ 0.999	0.603/ 0.999	< 0.001/ **0.002**

Data are presented as geometric means (95% confidence intervals) and analysed with linear mixed-effect model analysis. Data are from 24 OGTTs from 14 participants. *P* values are shown as non-adjusted/adjusted with significant adjusted *p* values in bold. Adjusted *p* values were calculated using the Bonferroni method

*NIL* Negative control, *MIT* Mitogen, *TB1* TB antigen tube 1, *TB2* TB antigen tube 2

Plasma glucose levels were not associated with IFN-γ-release for TB1 (β = 0.3, 95% CI -0.1 to 0.7, *p* = 0.999), TB2 (β = -0.1, 95% CI -0.4 to 0.3, *p* = 0.999) or MIT (β = 3.5, 95% CI -4.7 to 11.7, *p* = 0.999).

There was no systematic trend in the QFT test results during the OGTTs and at any timepoint 78–90% of the QFT tests were positive (Supplementary Table S1). In 6/24 (25%) OGTTs, one or more test results diverged from the baseline result. An overview of results from the OGTTs with diverging QFT test results is presented in Supplementary Table S2.

## Discussion

The main purpose of the present study was to investigate if an oral glucose load altered stimulated IFN-γ-release or QFT results in persons with TB or TBI. We did not observe an association between the prevailing plasma glucose levels at the QFT sampling timepoint and IFN-γ-release and glucose intake did not appear to affect the test results of the QFT. We did observe a borderline significant increase in IFN-γ-release at the end of the OGTT in the TB1 tube and a significant increase in the MIT tube, while the TB2 and NIL tubes were unaffected at all timepoints.

Only two studies have previously explored the impact of glucose on IFN-γ-release and report both a negative and positive association between the two [12, 21]. However, these studies differ significantly from ours as they utilised fasting plasma glucose measurements rather than repeated IGRAs during an OGTT. Additionally, fasting plasma glucose in these studies was primarily used to evaluate DM status rather than IFN-γ-release, and it remains unclear whether the QFT samples were collected together with the glucose measurements. It is therefore difficult to compare these studies to ours.

In the present study, the overall proportion of positive, negative and indeterminate QFT test results were stable during the OGTT (Supplementary Table S1). While studies on the impact of DM on IGRA test results are contradictive [11–15] our data support the reports which show no effect of DM on IGRA test performance [13–15]. We observed a variation of the QFT test results in 6/24 (25%) of the OGTTs, but these were seemingly at random and among participants with results around the cut-off points (Supplementary Table S2). Taken together, it is unlikely that glucose intake affects the QFT as a diagnostic test for TBI.

Stimulated IFN-γ-release increased after 240 min in the MIT tube, and to some extent in the TB1 tube, but was unaffected at all other timepoints. The small sample size in this study should be considered when assessing these results, especially regarding the TB1 tube where the statistical signal was less convincing. The more robust increase in the MIT tube does however suggest that there is a link between glucose intake and IFN-γ-release even though this does not appear to be mediated by the prevailing glucose levels. The underlying mechanism to this finding is unclear and we can only speculate on potential reasons. One explanation could be that the 10-h fast prior to the OGTT diminished T-cell reactivity which took at least 240 min to restore. A second possibility is that our results were affected by fluctuations in peripheral white blood cells (WBCs) which have been shown to decrease during 120 min long OGTTs [28, 29]. This means that our baseline QFTs might have contained more WBCs than QFTs taken later in the OGTT. It is not known when the number of WBCs return to baseline values after a glucose load and it is possible that the increased IFN-γ-release measured at minute 240 represents the return of T-cells to the circulation. A third explanation could be that we have captured the natural daytime variation of the immune system. The 240-min samples in the present study were taken just after noon but WBCs peak at midnight and reach their nadir at noon [30]. The effect of daytime variation should therefore, if anything, have a negative effect on IFN-γ-release at minute 240 in our study. A fourth explanation is that the results were caused by systematic errors in sample handling or analysis. But the tubes were handled identically and it is unlikely that a systematic error only affected two out of four tubes.

Limitations of this study include the small heterogeneous sample size with few TB patients, the low number of persons with DM and subsequently the low glucose levels during the OGTT. We are therefore unable to draw any firm conclusions regarding the effect of a glucose load on QFTs in persons with DM. The small sample size also prevents us from any meaningful analysis of potential differences between participants with TB and TBI. The lack of a placebo group is another limitation of this study which makes it difficult to ascertain if the results were caused by glucose intake or other factors. We also lack data on the number of circulating T-cells or WBCs at each sampling time point which would have given information on cell reactivity. However, strengths of the study are that we induced increased glucose levels in vivo (not in vitro) and that the sample handling, dilution and QFT analysis were performed by the same individual.

## Conclusion

Ingestion of an oral glucose load after a 10-h fast increased IFN-γ-release in the MIT tube of the QFT after 240 min. Plasma glucose levels at the QFT sampling timepoint were however not associated with IFN-γ-release and the test result of the QFT did not seem to be affected by glucose intake. Taken together, the overall effect of glucose intake on IGRAs is probably limited. Still, the role of glucose fluctuations in the interplay between DM and *Mtb* infections needs to be further elucidated in larger studies with a higher proportion of DM participants.

## Supplementary Information

Supplementary Material 1: Supplementary Table S1. Overall proportion of positive, negative and indeterminate QuantiFERON-TB Gold Plus test results from 24 OGTTs.

Supplementary Material 2: Supplementary Table S2. Data from the four participants with variation in their QuantiFERON-TB Gold Plus test results during the OGTTs.

Supplementary Material 3: Supplementary Figure S1. Flow diagram of included participants and OGTTs.

## Data Availability

The dataset used in the current study is available from the corresponding author on a reasonable request.

## Abbreviations

CI: Confidence interval
DM: Diabetes mellitus
ELISA: Enzyme-linked immunosorbent assay
HIV: Human immunodeficiency virus
IFN-γ: Interferon-γ
IGRA: Interferon-γ-release assay
MIT: Mitogen
Mtb: Mycobacterium tuberculosis
NIL: Negative control
OGTT: Oral glucose tolerance test
QFT: QuantiFERON-TB Gold Plus
TB: Tuberculosis disease
TBI: Tuberculosis infection
TB1: TB antigen tube 1
TB2: TB antigen tube 2
T-cell: T lymphocyte
WBC: White blood cell
