# Supplementary material for: The impact of an oral glucose load on IFN-γ-release in persons infected with Mycobacterium tuberculosis
Source: BMC Infect Dis. 2024 Sep 30;24:1079. doi: 10.1186/s12879-024-09920-x (PMC11443944; doi:10.1186/s12879-024-09920-x)
Supplement: Supplementary file 1 — Supplementary Material 1: Supplementary Table S1. Overall proportion of positive, negative and indeterminate QuantiFERON-TB Gold Plus test results from 24 OGTTs. [file 12879_2024_9920_MOESM1_ESM.docx]

**Supplementary Table S1.** Overall proportion of positive, negative and indeterminate results QuantiFERON-TB Gold plus results from 24 OGTTs.

| Time and number of participants | -15 min  n=23 | 60 min  n=20 | 90 min  n=22 | 120 min  n=22 | 240 min  n=23 |
| --- | --- | --- | --- | --- | --- |
| QFT positive (%) | 82,6% | 90,0% | 81,8% | 90,9% | 78,3% |
| QFT negative (%) | 4,3% | 5,0% | 4,5% | 0,0% | 8,7% |
| QFT indeterminate (%) | 13,0% | 5,0% | 13,6% | 9,1% | 13,0% |
| QFT, QuantiFERON-TB Gold plus | | | | | |
