# Supplementary material for: The impact of an oral glucose load on IFN-γ-release in persons infected with Mycobacterium tuberculosis
Source: BMC Infect Dis. 2024 Sep 30;24:1079. doi: 10.1186/s12879-024-09920-x (PMC11443944; doi:10.1186/s12879-024-09920-x)
Supplement: Supplementary file 2 — Supplementary Material 2: Supplementary Table S2. Data from the four participants with variation in their QuantiFERON-TB Gold Plus test results during the OGTTs. [file 12879_2024_9920_MOESM2_ESM.docx]

**Supplementary Table S2.** Results from the four participants with variation in their QFT net results during the OGTTs.

| **ID** | **Diagnosis** | **OGTT**  **No.** | **Timepoint (min)** | **TB1-NIL**  **(IU/mL)** | **TB2-NIL (IU/mL)** | **MIT-NIL (IU/mL)** | **NIL**  **(IU/mL)** | **Result** |
| --- | --- | --- | --- | --- | --- | --- | --- | --- |
|  |  |  |  |  |  |  |  |  |
| 1 | TBI | 1 | 15 | 0.85 | 0.56 | 7.61 | 2.34 | POSITIVE |
| 1 | TBI | 1 | 60 | Excluded since NIL was higher than TB1 or TB2 | | | | |
| 1 | TBI | 1 | 90 | 0.33 | 0.59 | 6.28 | 3.03 | NEGATIVE |
| 1 | TBI | 1 | 120 | 1.47 | 2.41 | >10 | 3.75 | POSITIVE |
| 1 | TBI | 1 | 240 | 2.77 | 3.02 | >10 | 2.04 | POSITIVE |
|  |  |  |  |  |  |  |  |  |
| 1 | TBI | 2 | 15 | Excluded since NIL was higher than TB1 or TB2 | | | | |
| 1 | TBI | 2 | 60 | 0.03 | 0.12 | >10 | 1.85 | NEGATIVE |
| 1 | TBI | 2 | 90 | 0.32 | 0.60 | >10 | 1.80 | POSITIVE |
| 1 | TBI | 2 | 120 | Excluded since NIL was higher than TB1 or TB2 | | | | |
| 1 | TBI | 2 | 240 | 0.25 | 0.02 | >10 | 1.84 | NEGATIVE |
|  |  |  |  |  |  |  |  |  |
| 2 | TBI | 2 | 15 | >10 | >10 | >10 | 8.22 | INDETERMINATE |
| 2 | TBI | 2 | 60 | >10 | >10 | >10 | 5.11 | POSITIVE |
| 2* | TBI* | 2* | 90* | 0.00* | 0.00* | 0.00* | >10* | INDETERMINATE* |
| 2 | TBI | 2 | 120 | Excluded since NIL was higher than TB1 or TB2 | | | | |
| 2 | TBI | 2 | 240 | >10 | >10 | >10 | 6.65 | POSITIVE |
|  |  |  |  |  |  |  |  |  |
| 3 | TBI | 1 | 15 | 0.33 | 0.34 | >10 | 0.11 | NEGATIVE |
| 3 | TBI | 1 | 60 | 0.38 | 0.57 | >10 | 0.07 | POSITIVE |
| 3 | TBI | 1 | 90 | 0.54 | 0.49 | >10 | 0.08 | POSITIVE |
| 3 | TBI | 1 | 120 | 0.53 | 0.54 | >10 | 0.10 | POSITIVE |
| 3 | TBI | 1 | 240 | 0.17 | 0.24 | >10 | 0.09 | NEGATIVE |
|  |  |  |  |  |  |  |  |  |
| 4 | TB | 1 | 15 | >10 | >10 | >10 | 8.19 | INDETERMINATE |
| 4 | TB | 1 | 60 | >10 | >10 | >10 | 4.61 | POSITIVE |
| 4 | TB | 1 | 90 | >10 | >10 | >10 | 5.64 | POSITIVE |
| 4 | TB | 1 | 120 | >10 | >10 | >10 | 5.39 | POSITIVE |
| 4 | TB | 1 | 240 | >10 | >10 | >10 | >10 | INDETERMINATE |
|  |  |  |  |  |  |  |  |  |
| 4 | TB | 2 | 15 | >10 | >10 | >10 | 2.90 | POSITIVE |
| 4 | TB | 2 | 60 | >10 | >10 | >10 | 5.50 | POSITIVE |
| 4 | TB | 2 | 90 | >10 | >10 | >10 | 8.67 | INDETERMINATE |
| 4 | TB | 2 | 120 | >10 | >10 | >10 | >10 | INDETERMINATE |
| 4 | TB | 2 | 240 | >10 | >10 | >10 | >10 | INDETERMINATE |
|  |  |  |  |  |  |  |  |  |
| *Nil was not higher than TB1 or TB2 after dilution and the samples were therefore not excluded.  TB, tuberculosis disease; TBI, tuberculosis infection; OGTT, oral glucose tolerance test; NIL, negative control; MIT, mitogen; TB1, TB antigen tube 1; TB2, TB antigen tube 2. | | | | | | | | |
