# Supplementary material for: The impact of an oral glucose load on IFN-γ-release in persons infected with Mycobacterium tuberculosis
Source: BMC Infect Dis. 2024 Sep 30;24:1079. doi: 10.1186/s12879-024-09920-x (PMC11443944; doi:10.1186/s12879-024-09920-x)
Supplement: Supplementary file 3 — Supplementary Material 3: Supplementary Figure S1. Flow diagram of included participants and OGTTs. [file 12879_2024_9920_MOESM3_ESM.docx]

**Supplementary Figure S1.** Flow diagram of included participants and OGTTs

**Reason for not participating in OGTT 1**:

Did not wish to participate n=33

**Reason for not participating in OGTT 2**:

Moved to another country: n=2

Did not wish to participate n=1

**Participants who participated in OGTT 2**

n=15

**Participants who participated in OGTT 1**

n=18

**Included in the two prospective studies**:

n=51

**Total number of OGTTs available for analysis of Interferon-γ-release**

n=33 (from n=18 participants)

**Reason for exclusion**

QFT were negative in 5/5 samples at OGTT 1 and OGTT 2: n= 4

Seroreverted in 5/5 samples at OGTT 2 n= 1

**Total number of OGTTs available for analysis**

n= 24 (from n=14 participants)
